# Supplementary material for: Effect of vaccination on household transmission of SARS-CoV-2 Delta variant of concern
Source: Nat Commun. 2022 Jun 30;13:3764. doi: 10.1038/s41467-022-31494-y (PMC9244879; doi:10.1038/s41467-022-31494-y)
Supplement: Supplementary file 1 — Supplementary Information [file 41467_2022_31494_MOESM1_ESM.pdf]

Supplementary Information

Effect of Vaccination on Household Transmission of SARS-CoV-2 Delta VOC

Frederik Plesner Lyngse[1,2,3,*]

Kåre Mølbak[3,4]

Matt Denwood[4]

Lasse Engbo Christiansen[5]

Camilla Holten Møller[3]

Morten Rasmussen[3]

Arieh Sierrah Cohen[3]

Marc Stegger[3]

Jannik Fonager[3]

Raphael Sieber[3]

Kirsten Ellegaard[3]

Claus Nielsen[3]

Carsten Thure Kirkeby[4]

[*]Correspondence to Frederik Plesner Lyngse, fpl@econ.ku.dk. Affiliations: [1]Department of Economics & Center for Economic Behaviour and Inequality, University of Copenhagen, Copenhagen, Denmark; [2]Danish Ministry of Health, Copenhagen, Denmark; [3]Statens Serum Institut, Copenhagen, Denmark; [4]Department of Veterinary and Animal Sciences, Faculty of Health and Medical Sciences, University of Copenhagen, Copenhagen, Denmark.; [5]Department of Applied Mathematics and Computer Science, Dynamical Systems, Technical University of Denmark, Richard Petersens Plads, 324, DK-2800 Kgs. Lyngby, Denmark.

S1 Background

This section provides some background statistics on the SARS-CoV-2 pandemic situation in Denmark, between June and November, 2021.

Figure S1 shows the number of tests performed per thousand Danish citizens (green, left axis) and the number of new positive cases identified per million Danish citizens (purple, right axis) between June and November, 2021.

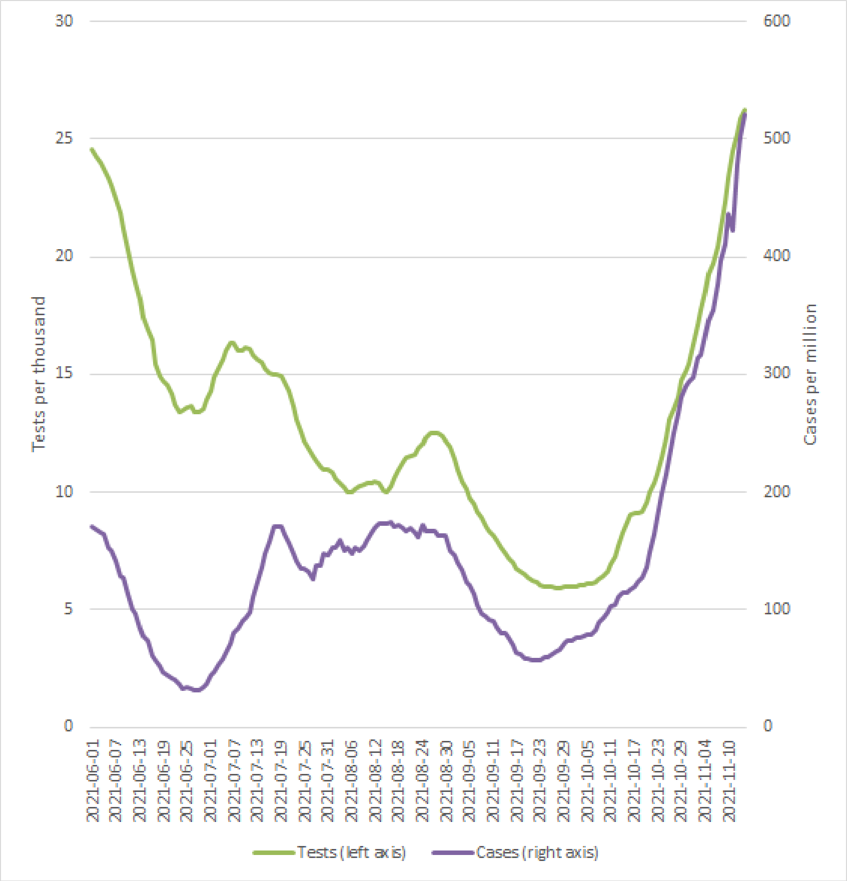

Figure S1: Tests and Cases

Notes: This figure shows the number of tests performed per thousand Danish citizens (green, left axis) and the number of new positive cases identified per million Danish citizens (purple, right axis), June to November, 2021. Data source: Statens Serum Instutit[24].

Between June and July the Delta VOC became the dominant variant in Denmark, reaching approximately 100% of all new cases (Figure S2).

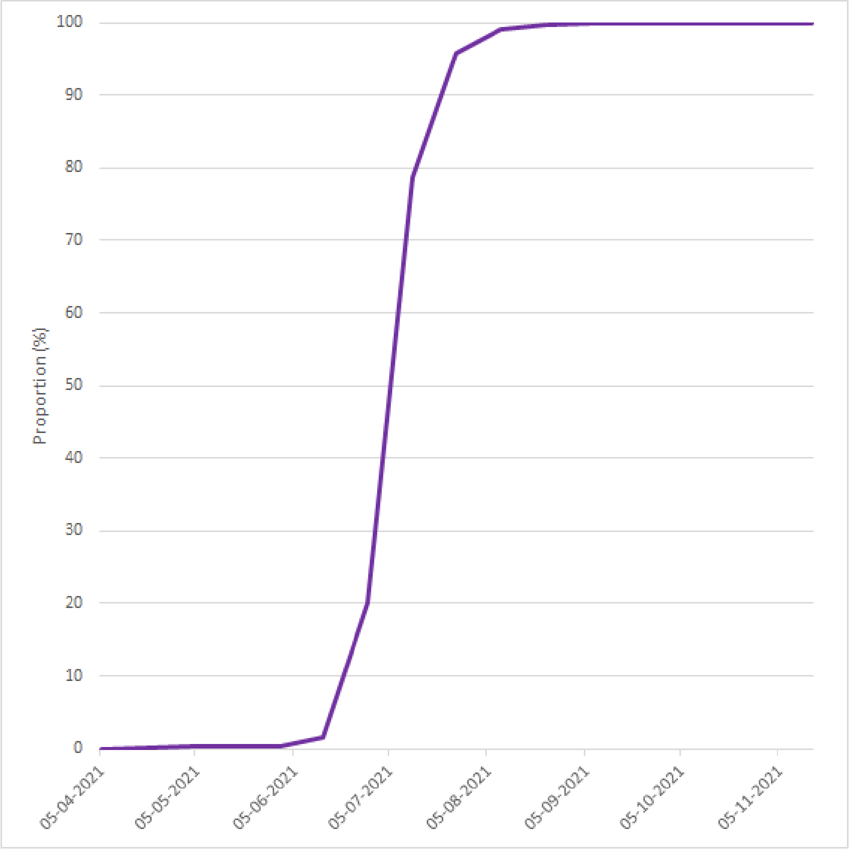

Figure S2: Proportion of Cases with Delta VOC

Notes: This figure shows the proportion of sequenced positive samples that were identified as the Delta VOC in Denmark, April to November, 2021. Data source: Our World in Data[25].

In the study period, vaccinations were rolled out, going from below 40% fully vaccinated to more than 75% fully vaccinated individuals (Figure S3).

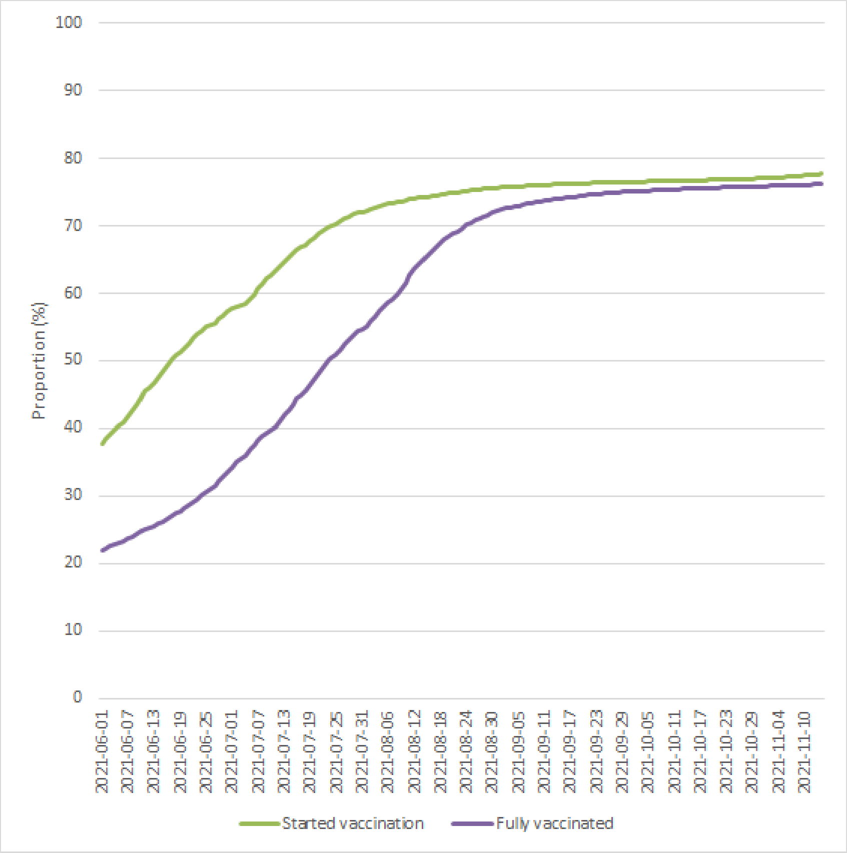

Figure S3: Proportion of population vaccinated

Notes: This figure shows the proportion of the population partially and fully vaccinated, June to November 2021. Data source: Statens Serum Instutit[26].

Denmark had a vaccination roll-out strategy that prioritized vulnerable people. Nursery home residents had highest prioritization (group 01), followed by citizens above age 65 who had the need of personal help and care (group 02), then older people above age 85 (group 03). The next three groups (04-06) contained employees in healthcare and social work, high-risk patients, and relatives of high-risk patients. The remaining population was subsequently prioritized based on birth year (groups 07-17). Naturally, this leads to a correlation between calendar time and vaccination status conditional on age. Figure S4 shows the proportion of each group being fully vaccinated from January to November, 2021. We see that the compliance is generally high, and fast roll-out is seen in all groups. This roll-out strategy also meant that most of the vulnerable population was fully vacci-

nated, when the Delta VOC was first detected in Denmark. For example, more than 90% of individuals above age 65 (group 09) were fully vaccinated.

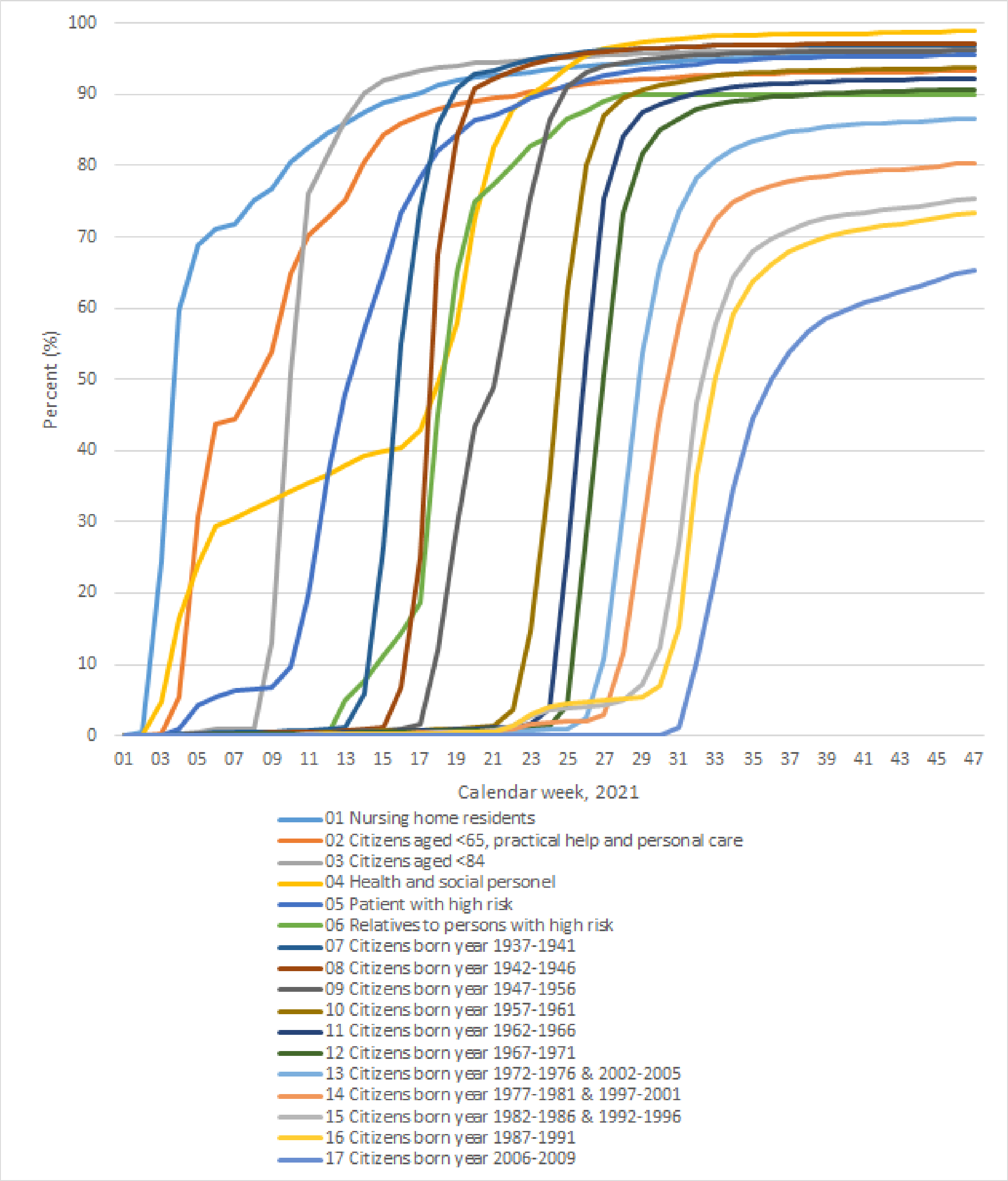

Figure S4: Vaccination roll-out, proportion of vaccination group fully vaccinated

Notes: This figure shows the proportion of the vaccination groups that are fully vaccinated by calendar week, January to November, 2021. Data source: Statens Serum Instutit [26].

Finally, Denmark had relatively lenient restrictions during our study period. Figure S5 shows the government stringency index between June and November, 2021. The index is a composite measure of the strictness of policy responses and is based on nine response indicators including school closures, workplace closures, and travel bans. The index is calculated by the Oxford COVID-19 Government Response Tracker.

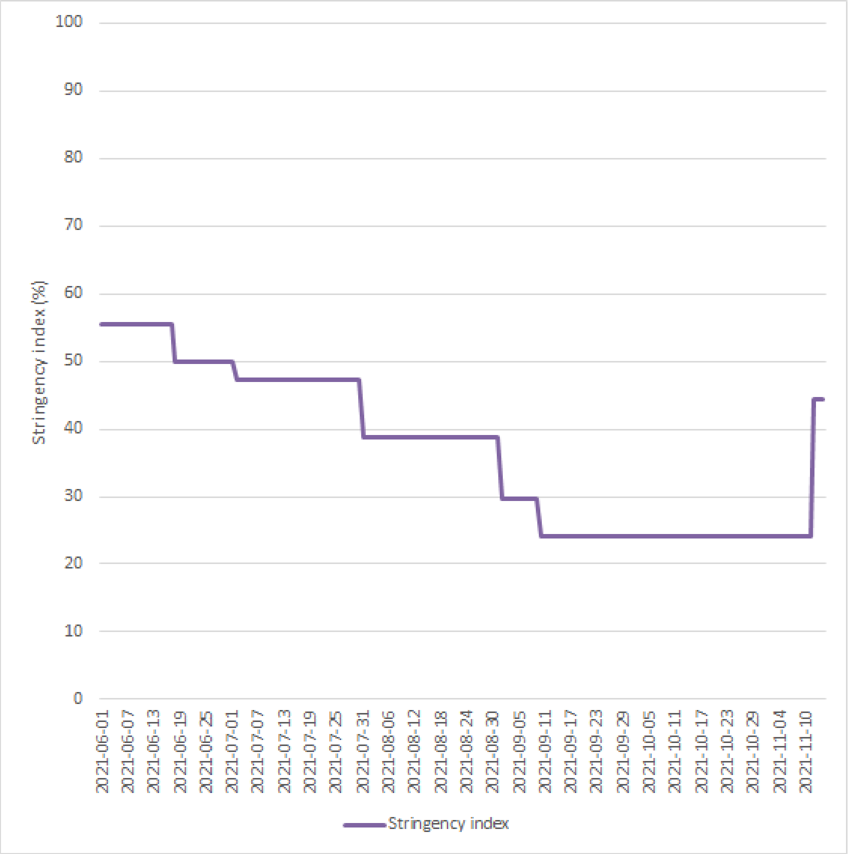

Figure S5: Government stringency index

Notes: This figure shows the government stringency index June to November, 2021. The index is a composite measure of the strictness of policy responses and is based on nine response indicators including school closures, workplace closures, and travel bans. The index is calculated by the Oxford COVID-19 Government Response Tracker. Data source: Our World in Data[25].

S1.1 Danish testing program

The Danish SARS-CoV-2 testing program is based on easily accessible testing that is free of charge. The use of testing has been broadly accepted by the public and by December 2021, more than 15% of the Danish population were RT-PCR tested each week and a similar percentage were antigen tested. Self-administered home tests only became widely used after our study period, by December 2021. There are three tracks in the testing

program: The healthcare track; the community track; and the rapid test track. Both the healthcare track and the community track are based on RT-PCR detection of SARS-CoV-2 in oropharyngeal swabs, whereas the rapid test track is based on antigen detection. The RT-PCR results are usually reported within 24 hours. Individuals that tested positive in the rapid test track were recommended to take a follow up RT-PCR test to confirm the test result. All positive test results are registered in the Danish national microbiology database (MiBa), which thus provides a comprehensive overview of the pandemic on a national level.

S1.2 Genomic data

As part of the public pandemic response and surveillance strategy, all positive RT-PCR samples in the study period were selected for whole-genome sequencing by The Danish COVID-19 Genome Consortium[27] if they showed a Ct value below 35[20]. Whole-genome sequencing of the positive samples was performed on short read Illumina technology (Illumina, San Diego, California, United States) with the Illumina COVIDSeq Test kit (Illumina, San Diego, California, United States). The library preparation was performed as described by the manufacturer with a few modifications: COVIDSeq primer pool 2 was spiked with 1x amplicon 64 primer pair and 0.2x amplicon 70 and 74 primer pairs from the ARTIC v3 amplicon sequencing panel[28]. Furthermore, in the cDNA amplification, the denaturation was adjusted to 95°C for 15 seconds and annealing at 63°C for 5 min. Samples were pooled and sequenced in batches for 384 on the Illumina NextSeq 500/550 using the mid-output kit v2.5 (150 cycles) kit, or 2x384 on the Illumina NovaSeq 6000 using the SP reagent kit (Illumina, San Diego, California, United States). Consensus sequences were called using "IVAR consensus", with an in-house implementation of IVAR (version 1.3.1). The resulting consensus sequences were considered for variant calling when containing <3,000 ambiguous sites including N's. Variants were called using Pangolin (version 3.1.16) with PangoLEARN assignment algorithm on the consensus sequences[29].

S2 Additional Analyses

Table S1: Summary Statistics, stratified by primary case level

	Fully vaccinated				Unvaccinated			
	Primary Cases	Household Contacts	Secondary Cases	SAR (%)	Primary Cases	Household Contacts	Secondary Cases	SAR (%)
**Total**	8,262	15,248	3,069	20	16,431	38,336	8,562	22
**Sex**								
Male	4,001	7,209	1,562	22	8,301	19,279	4,075	21
Female	4,261	8,039	1,507	19	8,130	19,057	4,487	24
**Age**								
0-10	0	0	0	-	3,668	9,963	2,110	21
10-20	721	1,731	97	6	4,859	12,351	2,150	17
20-30	1,368	2,271	199	9	4,131	7,584	1,436	19
30-40	1,131	2,619	671	26	2,316	5,274	1,813	34
40-50	1,676	3,980	798	20	923	2,224	798	36
50-60	1,601	2,597	564	22	407	766	198	26
60-70	1,085	1,304	382	29	109	154	49	32
70-80	680	746	358	48	18	20	8	40
**Household Size**								
2	3,859	3,859	1,110	29	3,704	3,704	911	25
3	1,809	3,336	520	16	4,092	7,456	1,584	21
4	1,785	4,963	879	18	5,080	13,699	3,126	23
5	643	2,320	430	19	2,762	9,920	2,171	22
6	166	770	130	17	793	3,557	770	22
**Vaccination**								
AstraZeneca	514	1,015	204	20	-	-	-	-
Janssen	529	879	163	19	-	-	-	-
Moderna	366	689	115	17	-	-	-	-
Pfizer	6,853	12,665	2,587	20	-	-	-	-

Notes: Household contacts and secondary cases are grouped based on their associated primary case characteristics. See Table 1 for contacts and secondary cases grouped by their own characteristics. The secondary attack rate (SAR) is expressed in percentages.

S2.1 Age-by-age transmission

To investigate the age-related transmission patterns, we split the data into 20-year age groups of both the primary cases and household contacts and estimated the secondary attack rate (SAR) between all combinations of age groups, stratified by vaccination status of both the primary case and household contacts (Figure S6). Generally, the SAR was highest, when both the primary case and household contacts were unvaccinated (top-left panel). Furthermore, the SAR was generally higher when the primary case was vaccinated and the contacts was not vaccinated (bottom left panel), than when the opposite was

true (top right panel), showing that vaccination overall has a higher VE_S than VE_I, corroborating the findings in Table 2.

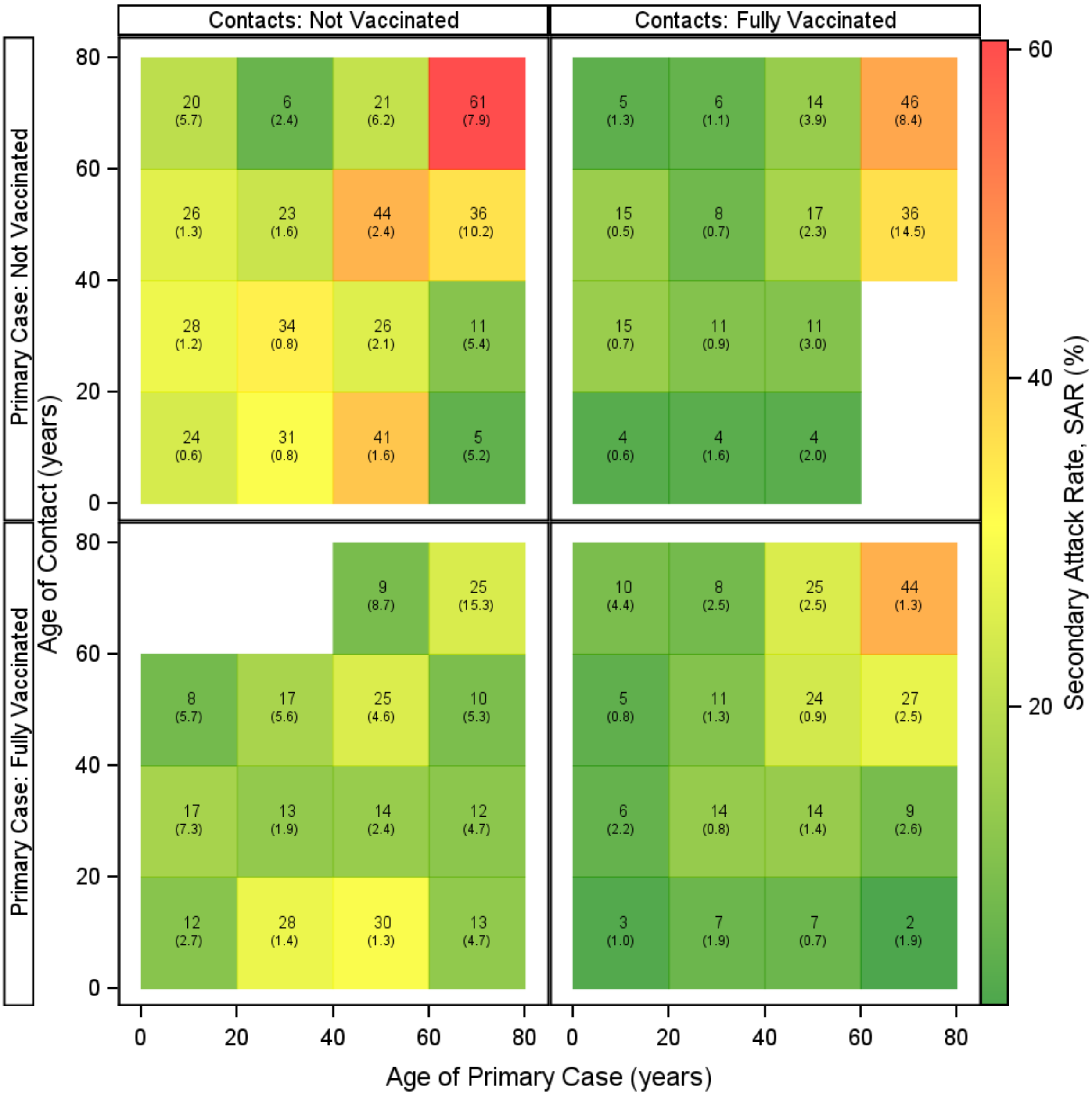

Figure S6: Secondary Attack Rate, age-by-age, stratified by vaccination status

Notes: This figure presents secondary attack rates (SAR) stratified by the age of the primary case and household contacts, and stratified by the vaccination status of the primary case and household contacts. Numbers show the estimated SAR with standard errors clustered on the household level in parentheses. Table S2 provides number of observations used to estimate the SAR for each combination.

Table S2: Number of observations for Figure S6

Primary case: Not vaccinated \Contact: Not Vaccinated				
**Age of contact (years)**				
**60-80**	[12/59/54]	[7/108/99]	[9/42/40]	[23/38/38]
**40-60**	[402/1,520/1,230]	[180/800/671]	[205/471/446]	[9/25/22]
**20-40**	[484/1,704/1,373]	[1,312/3,906/3,357]	[121/469/394]	[<5/35/27]
**0-20**	[1,840/7,540/5,519]	[1,482/4,850/2,937]	[587/1,444/803]	[<5/19/17]
	**0-20**	**20-40**	**40-60**	**60-80**
	**Age of primary case (years)**			

Primary case: Fully vaccinated \Contact: Not Vaccinated				
**Age of contact (years)**				
**60-80**			[<5/11/11]	[<5/8/8]
**40-60**	[<5/24/20]	[8/47/44]	[23/91/89]	[<5/31/30]
**20-40**	[<5/24/23]	[47/352/307]	[33/231/203]	[6/49/44]
**0-20**	[19/163/135]	[465/1,653/955]	[516/1,699/1,106]	[8/61/42]
	**0-20**	**20-40**	**40-60**	**60-80**
	**Age of primary case (years)**			

Primary case: Not vaccinated \Contact: Fully Vaccinated				
**Age of contact (years)**				
**60-80**	[13/280/241]	[28/470/384]	[13/91/78]	[16/35/35]
**40-60**	[993/6,742/4,598]	[112/1,457/1,090]	[45/267/253]	[<5/11/11]
**20-40**	[471/3,232/2,408]	[122/1,116/988]	[12/109/99]	
**0-20**	[45/1,237/1,072]	[6/151/144]	[<5/97/86]	
	**0-20**	**20-40**	**40-60**	**60-80**
	**Age of primary case (years)**			

Primary case: Fully vaccinated \Contact: Fully Vaccinated				
**Age of contact (years)**				
**60-80**	[<5/42/35]	[12/145/115]	[79/315/300]	[620/1,402/1,391]
**40-60**	[53/1,070/639]	[76/696/520]	[524/2,202/2,166]	[89/324/311]
**20-40**	[7/120/108]	[247/1,769/1,523]	[84/590/524]	[11/122/103]
**0-20**	[8/288/254]	[15/216/182]	[102/1,438/1,073]	[<5/53/46]
	**0-20**	**20-40**	**40-60**	**60-80**
	**Age of primary case (years)**			

Notes: This table provides number of observations used for estimating the SAR stratified by age and vaccination status in Figure S6. [./././] Number of secondary cases / number of contacts / number of primary cases.

S2.2 Intra-household correlation of lineages

We found an overall intra-household correlation of SARS-CoV-2 lineages of 88% (95%-CI: 87-89) (Table S3), i.e., 88% of all secondary cases had the same sublineage of Delta as the primary case within the same household (conditional on the secondary test sample having a successfully sequenced genome). This intra-household correlation is lower compared to the one found in, e.g., Lyngse et al.[20], at 96-99%. This could be because the society is more open, so there is a higher risk of community infection relative to household infection. However, it could also be due to uncertainty in the classification of subtype lineages of the Delta VOC, which is changing over time. In the present study, we used PANGO Lineage classifications from 2021-11-10, why bias from temporal differences in the PANGO classifications were not an issue.

In the present study, there is potential bias in the intra-household correlation of lineages across vaccinated and unvaccinated individuals, which could invalidate our comparisons of the relative risks. Table S3 presents the intra-household correlation of lineages across all combinations of vaccinated and unvaccinated primary and secondary cases. We found no statistically significant difference across any of the combinations, so there is no evidence for differential bias across our groups of comparison.

Table S3: Intra-household correlation of lineages

	**Secondary Case**		
	**Pool**	**Vaccinated**	**Unvaccinated**
**Primary Case**			
Pool	88	89	88
	(87-89)	(88-90)	(87-89)
	[9,580/6,852]	[3,156/2,860]	[6,424/4,652]
Vaccinated	90	89	90
	(88-91)	(87-91)	(88-92)
	[2,443/1,980]	[1,573/1,499]	[870/656]
Unvaccinated	88	89	87
	(87-89)	(87-90)	(86-88)
	[7,137/4,872]	[1,583/1,361]	[5,554/3,996]

Notes: This table provides estimates on the intra-household correlation of lineages, i.e., the probability that the secondary case had the same subtype lineage as the primary case. 95%-confidence intervals clustered on the household level presented in parenthesis. Number of observations and household clusters for each point estimate are presented in hard brackets: [Number of observations/number of households].

S2.3 Correlation of vaccination status within households

Vaccination status among household members are likely correlated due to several reasons. First, individuals are probably likely to live with other individuals with the same beliefs. Thus individuals that are pro being vaccinated are more likely to live together with other individuals that also are pro being vaccinated—and vice versa. Second, individuals are likely to live with a partner around their own age. As vaccination eligibility is correlated with age, individuals are likely to be eligible for vaccination around the same time. Third, there may be a fixed cost of being vaccinated, e.g., transportation time to the vaccination place. Thus, households may pool their time of vaccination on the same day to minimize these costs.

Figure S7 provides estimates of the correlation of the vaccination status between primary cases and contacts, stratified by ten year age groups. It clearly shows some correlation among household members. Among young children (<12 years) there is perfect correlation, as they were not eligible for being vaccinated. We found a correlation of 0.63 within the full sample and a correlation of 0.72, when restricting the sample to individuals above age 12.

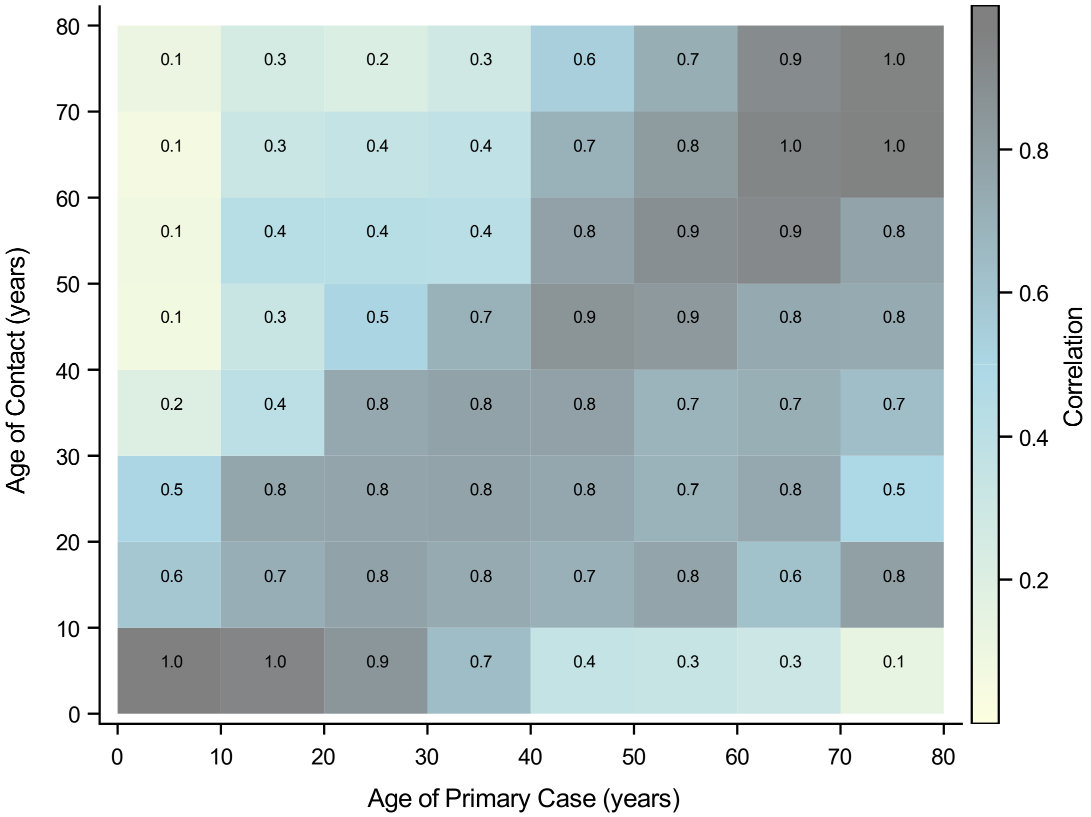

Figure S7: Correlation of vaccination status within households

Notes: This figures provides estimates of the correlation of the vaccination status between primary cases and household contacts, stratified by ten-year age groups.

S2.4 Testing probabilities across vaccinated and unvaccinated individuals

When estimating the probability that an individual tests positive, it is conditional on the individual actually being tested. Selection bias is a potential concern, if the vaccination status of a household contact within the household is correlated with the probability of being tested after the identification of the primary case. Overall, we find that household contacts that are fully vaccinated are 7 percentage points (on a basis of 75%) more likely to be tested 1-14 days after exposure, compared to unvaccinated contacts (Table S8.a). This suggests that there is a correlation between the vaccination status of the exposed contact and the compliance with being tested after exposure to a household primary case. Panel (b) shows there are temporal differences in the propensity to be tested. These

differences are highly correlated with the time of the school summer holiday. Panel (c) shows that there are differences across age groups in the propensity to be tested. Overall, we found that fully vaccinated household contacts were more likely to be tested after exposure to a primary case compared to unvaccinated contacts.

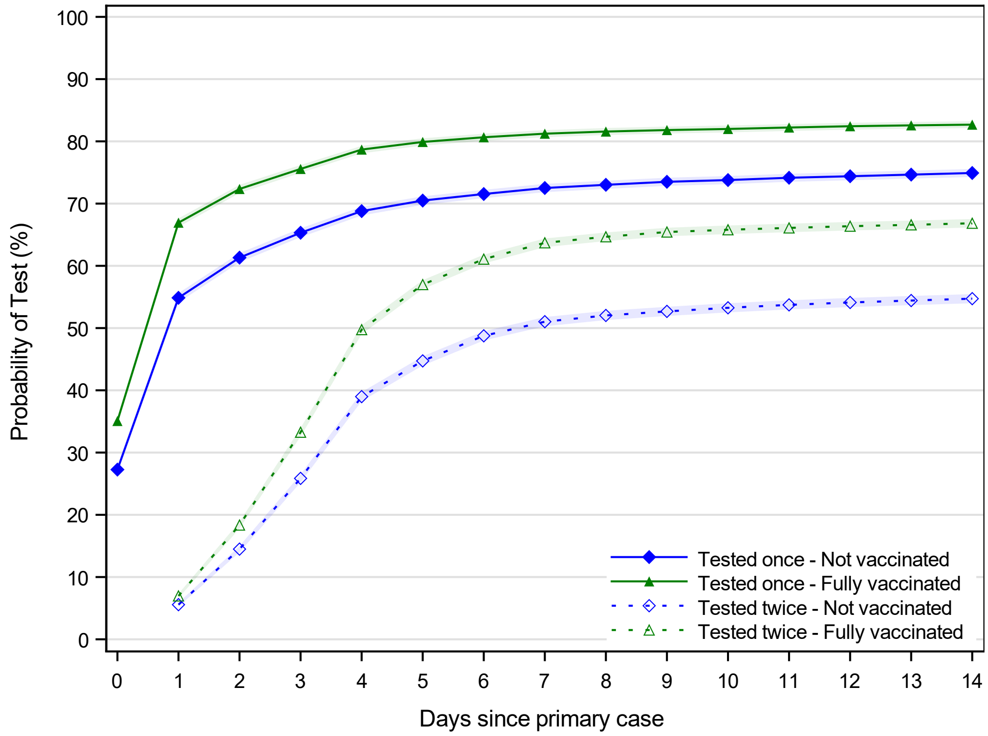

(a) Overall testing propensity of first and second test

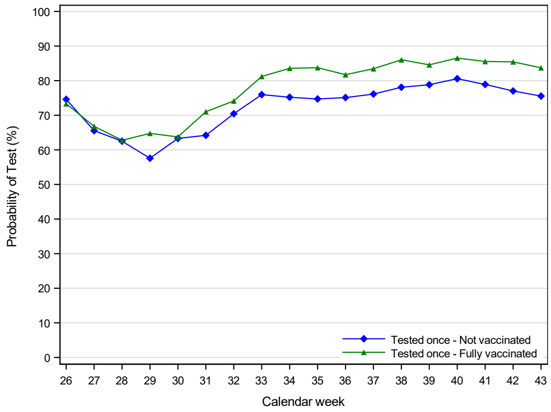

(b) By calendar week

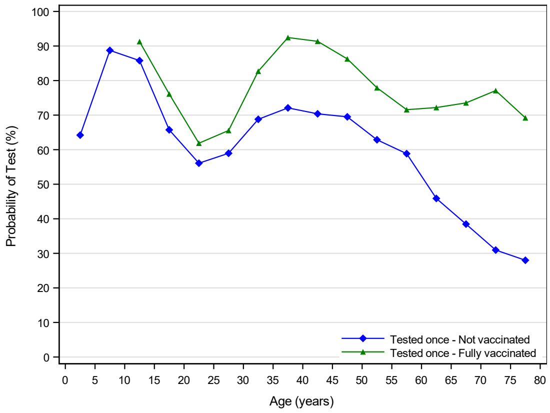

(c) By age of household contact

Figure S8: Probability of being tested after household exposure

Notes: This figure provides estimates of the difference in the probability that fully vaccinated household contacts have been tested after identification of a primary case compared with unvaccinated contacts. Panel (a) shows the probability of being tested once (solid lines) and tested twice (dotted lines). It shows that the household contacts are exhibiting the same trend in testing since exposure, unconditional of the vaccination status. Vaccinated contacts have a significantly higher rate of being tested after diagnosis of a primary case in the same household. Panel (b) shows the testing propensity for being tested once within 14 days, stratified by vaccination status and calendar week (defined by primary case sample date). Panel (c) shows the testing propensity, stratified by age of the household contact. Markers present point estimates, while shaded areas are 95%-confidence intervals clustered on the household level.

S2.5 Ct values for vaccinated vs unvaccinated cases

One concern in investigating the infectiousness among vaccinated and unvaccinated cases is that the viral load may differ across the two groups. Indeed the literature has shown that the viral load can be reduced for vaccinated individuals with breakthrough infections compared to unvaccinated individuals with infections[16]. We found that samples from vaccinated primary cases had a lower viral load distribution (higher Ct values) compared to samples from unvaccinated primary cases (Figure S9).

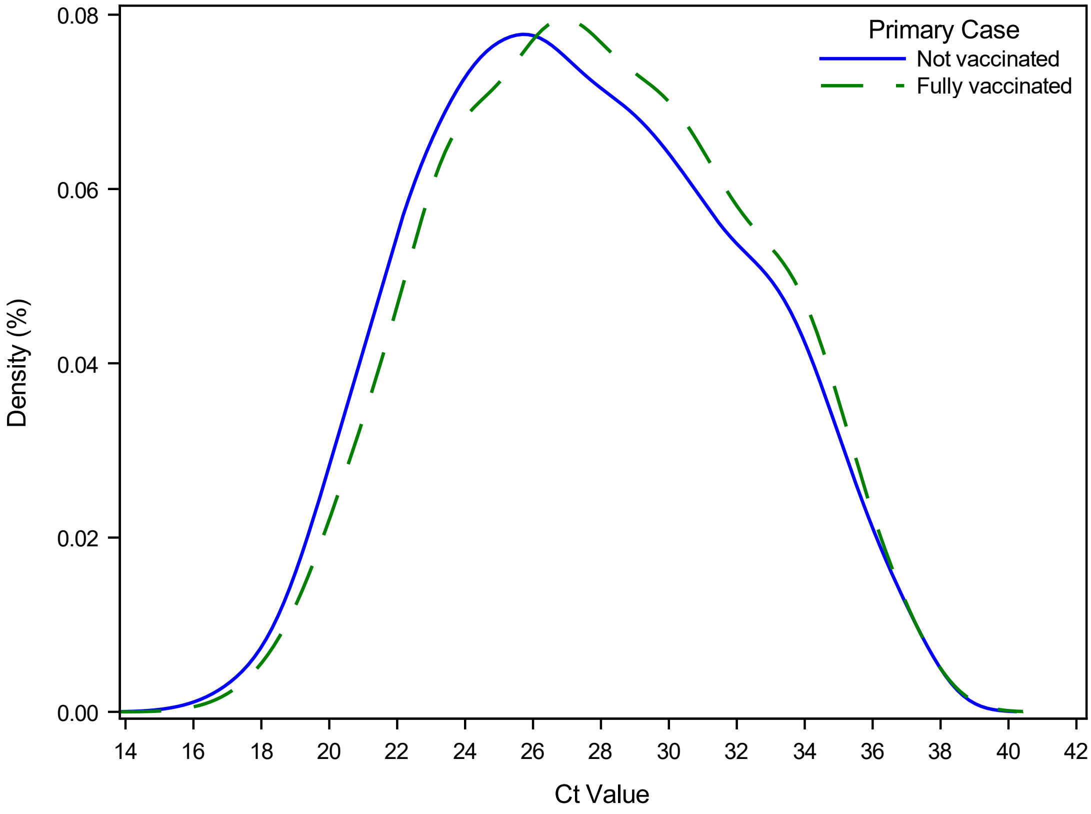

Figure S9: Ct values across vaccinated and unvaccinated primary cases

Notes: This figure shows the density of the Ct values of primary cases stratified by the vaccination status. Samples from vaccinated cases had a lower viral load (higher Ct values) distribution compared to samples from unvaccinated primary cases.

However, this result may be biased if, for example, vaccinated cases with a breakthrough infection are being diagnosed later in their infection compared to unvaccinated cases. This could for example occur if vaccinated individuals are tested less regularly than unvacci-

nated individuals, if they are asymptomatic or do not develop as severe symptoms, or if they believe they are less likely to be infected because of their vaccination status. We investigated this potential selection bias by comparing secondary cases that were fully vaccinated with unvaccinated secondary cases that tested positive on the same day after exposure of the primary case (Figure 2 and Table S4). We found that unvaccinated secondary cases had a 1.6 point lower Ct value, which translates to a 3-fold higher viral load due to the doubling property of the Ct measurement ($2^{1.6} = 3$) (Table S4, model II).

Table S4: Comparison of Ct values between vaccinated and unvaccinated secondary cases

	**I**	**II**	**III**
Fully vaccinated	1.61	1.63	1.30
	(0.1651)	(0.1843)	(0.2458)
Days since primary case, fixed effects	YES	YES	YES
Age, fixed effects	YES	YES	YES
Age, primary case, fixed effects	NO	YES	YES
Fully vaccinated, primary case, fixed effects	NO	YES	YES
Ct value, primary case, fixed effects	NO	NO	YES
N Observations	6,938	6,938	4,091
N households	5,088	5,088	2,963

Notes: This table provides regression estimates for the sample Ct value for secondary household cases, comparing unvaccinated secondary cases with vaccinated secondary cases, controlling for the day of testing since exposure to the primary case. The regression estimates from three separate model specifications are presented in column I, II, and III. Standard errors clustered on the household level in parenthesis.

S2.6 Robustness of VE estimates

This section addresses the robustness of the VE estimates presented in Table 2. Table S5 provides VE estimates, conditional on the household contact having a test result. Thus, we do not assume that all untested contacts are negative. See Appendix section S2.4 for differences in the probability of being tested across vaccinated and unvaccinated household contacts. Table S6 provides VE estimates, controlling for sample Ct values of the primary case as a proxy for viral load. Table S7 provides VE estimates, controlling for sample Ct values of the primary case as a proxy for viral load, conditional on the household contact having a test result. Table S8 provides VE estimates stratified by time since vaccination, in order to take waning immunity into account. Table S9 provides VE estimates stratified

by time since vaccination, in order to take waning immunity into account, conditional on the contacts case having a test result.

Table S5: Vaccine effectiveness, conditional on household contacts having a test result

	**Susceptibility**			**Infectiousness**			**Total**
Primary cases vaccinated	**Pool**	**Not**	**Fully**				
Household contacts vaccinated				**Pool**	**Not**	**Fully**	
Estimator	$VE_{S(\cdot,V/\cdot,N)}$	$VE_{S(N,V/N,N)}$	$VE_{S(V,V/V,N)}$	$VE_{I(V,\cdot/N,\cdot)}$	$VE_{I(V,N/N,N)}$	$VE_{I(V,V/N,V)}$	$VE_{T(V,V/N,N)}$
VE (%)	65	61	61	51	36	28	72
(95%-CI)	(64;67)	(59;64)	(56;65)	(48;53)	(32;40)	(20;35)	(70;73)
Age FE	YES	YES	YES	YES	YES	YES	YES
Week FE	YES	YES	YES	YES	YES	YES	YES
Female	YES	YES	YES	YES	YES	YES	YES
Household size FE	YES	YES	YES	YES	YES	YES	YES
Female, primary case	YES	YES	YES	YES	YES	YES	YES
Age, primary case, FE	YES	YES	YES	YES	YES	YES	YES
Ct value, primary case, FE	NO	NO	NO	NO	NO	NO	NO
Conditional on test	YES	YES	YES	YES	YES	YES	YES
N Observations	42,170	29,472	12,698	42,170	20,592	21,578	26,310
N Households	21,086	13,698	7,388	21,086	12,744	14,201	17,220

Notes: This table provides VE estimates from the same model as Table 2, conditional on the household contacts having a test result. This table provides estimates of vaccine effectiveness (%) against susceptibility (VE_S) as a pooled estimate ("Pool") as well as stratified by whether the primary case was unvaccinated ("Not") or fully vaccinated ("Fully"). The estimates of vaccine effectiveness against infectiousness (VE_I) is given as a pooled estimate and stratified by the vaccination status of the contacts within the household. The total vaccine effectiveness (VE_T) is defined as both the primary case and contacts being vaccinated relative to them both being unvaccinated. Note that the VE estimates across columns are not directly comparable as they are estimated on stratified samples. 95% confidence intervals clustered on the household level in parentheses. FE = included as fixed effects in the model.

Table S6: Vaccine effectiveness, controlling for primary case sample Ct values

	Susceptibility			Infectiousness			Total
Primary cases vaccinated	**Pool**	**Not**	**Fully**				
Household contacts vaccinated				**Pool**	**Not**	**Fully**	
Estimator	$VE_{S(\cdot,V/\cdot,N)}$	$VE_{S(N,V/N,N)}$	$VE_{S(V,V/V,N)}$	$VE_{I(V,\cdot/N,\cdot)}$	$VE_{I(V,N/N,N)}$	$VE_{I(V,V/N,V)}$	$VE_{T(V,V/N,N)}$
VE (%)	61	62	47	40	30	4	66
(95%-CI)	(58;63)	(58;64)	(38;54)	(35;45)	(23;36)	(-10;17)	(62;69)
Age FE	YES	YES	YES	YES	YES	YES	YES
Week FE	YES	YES	YES	YES	YES	YES	YES
Female	YES	YES	YES	YES	YES	YES	YES
Household size FE	YES	YES	YES	YES	YES	YES	YES
Female, primary case	YES	YES	YES	YES	YES	YES	YES
Age, primary case, FE	YES	YES	YES	YES	YES	YES	YES
Ct value, primary case, FE	YES	YES	YES	YES	YES	YES	YES
Conditional on test	NO	NO	NO	NO	NO	NO	NO
N Observations	28,364	20,148	7,917	28,364	14,236	14,128	17,564
N Households	13,056	8,577	4,311	13,056	8,162	8,898	10,627

Notes: This table provides VE estimates from the same model as Table 2, controlling for the sample Ct value of the primary case. This table provides estimates of vaccine effectiveness (%) against susceptibility (VE_S) as a pooled estimate ("Pool") as well as stratified by whether the primary case was unvaccinated ("Not") or fully vaccinated ("Fully"). The estimates of vaccine effectiveness against infectiousness (VE_I) is given as a pooled estimate and stratified by the vaccination status of the contacts within the household. The total vaccine effectiveness (VE_T) is defined as both the primary case and contacts being vaccinated relative to them both being unvaccinated. Note that the VE estimates across columns are not directly comparable as they are estimated on stratified samples. 95% confidence intervals clustered on the household level in parentheses. FE = included as fixed effects in the model. VE_S for primary cases fully vaccinated only includes primary cases with a sample Ct value >20.

Table S7: Vaccine effectiveness, controlling for primary case Ct values, conditional on household contacts having a test result

	**Susceptibility**			**Infectiousness**			**Total**
Primary cases vaccinated	**Pool**	**Not**	**Fully**				
Household contacts vaccinated				**Pool**	**Not**	**Fully**	
Estimator	$VE_{S(\cdot,V/\cdot,N)}$	$VE_{S(N,V/N,N)}$	$VE_{S(V,V/V,N)}$	$VE_{I(V,\cdot/N,\cdot)}$	$VE_{I(V,N/N,N)}$	$VE_{I(V,V/N,V)}$	$VE_{T(V,V/N,N)}$
VE (%)	65	62	62	49	34	24	71
(95%-CI)	(63;67)	(59;65)	(56;68)	(45;52)	(27;39)	(12;34)	(68;73)
Age FE	YES	YES	YES	YES	YES	YES	YES
Week FE	YES	YES	YES	YES	YES	YES	YES
Female	YES	YES	YES	YES	YES	YES	YES
Household size FE	YES	YES	YES	YES	YES	YES	YES
Female, primary case	YES	YES	YES	YES	YES	YES	YES
Age, primary case, FE	YES	YES	YES	YES	YES	YES	YES
Ct value, primary case, FE	YES	YES	YES	YES	YES	YES	YES
Conditional on test	YES	YES	YES	YES	YES	YES	YES
N Observations	22,638	15,767	6,627	22,638	10,802	11,836	13,865
N Households	11,253	7,241	3,861	11,253	6,757	7,753	9,158

Notes: This table provides VE estimates from the same model as Table 2, controlling for the sample Ct value of the primary case, conditional on the household contacts having a test result. This table provides estimates of vaccine effectiveness (%) against susceptibility (VE_S) as a pooled estimate ("Pool") as well as stratified by whether the primary case was unvaccinated ("Not") or fully vaccinated ("Fully"). The estimates of vaccine effectiveness against infectiousness (VE_I) is given as a pooled estimate and stratified by the vaccination status of the contacts within the household. The total vaccine effectiveness (VE_T) is defined as both the primary case and contacts being vaccinated relative to them both being unvaccinated. Note that the VE estimates across columns are not directly comparable as they are estimated on stratified samples. 95% confidence intervals clustered on the household level in parentheses. FE = included as fixed effects in the model. VES for primary cases fully vaccinated only includes primary cases with a sample Ct value >20.

Table S8: Vaccine effectiveness by time since vaccination

	Susceptibility			Infectiousness		
Primary cases vaccinated	**Pool**	**Not**	**Fully**			
Household contacts vaccinated				**Pool**	**Not**	**Fully**
Estimator	$VE_{S(\cdot,V/\cdot,N)}$	$VE_{S(N,V/N,N)}$	$VE_{S(V,V/V,N)}$	$VE_{I(V,\cdot/N,\cdot)}$	$VE_{I(V,N/N,N)}$	$VE_{I(V,V/N,V)}$
Time since vaccination						
0-1 months	71	69	59	57	42	36
	(69;72)	(67;71)	(53;64)	(53;61)	(34;48)	(26;44)
2-3 months	49	50	32	35	26	-8
	(46;52)	(45;54)	(23;39)	(30;39)	(19;33)	(-21;4)
4-5 months	38	42	26	28	22	-8
	(32;43)	(32;51)	(14;37)	(20;35)	(6;36)	(-25;7)
5-6 months	27	24	11	23	16	-29
	(18;35)	(11;36)	(-8;27)	(13;32)	(-2;31)	(-52;-9)
7-8 months	32	26	28	29	17	-18
	(16;45)	(1;45)	(1;48)	(14;41)	(-6;35)	(-50;7)
Age FE	YES	YES	YES	YES	YES	YES
Week FE	YES	YES	YES	YES	YES	YES
Female	YES	YES	YES	YES	YES	YES
Household size FE	YES	YES	YES	YES	YES	YES
Female, primary case	YES	YES	YES	YES	YES	YES
Age, primary case, FE	YES	YES	YES	YES	YES	YES
Ct value, primary case, FE	NO	NO	NO	NO	NO	NO
Conditional on test	NO	NO	NO	NO	NO	NO
N Observations	53,584	38,336	15,248	53,584	27,486	26,098
N Households	24,693	16,431	8,262	24,693	15,559	16,493

Notes: This table provides VE estimates from the same model as Table 2 by time since vaccination (bi-monthly). This table provides estimates of vaccine effectiveness (%) against susceptibility (VE_S) as a pooled estimate ("Pool") as well as stratified by whether the primary case was unvaccinated ("Not") or fully vaccinated ("Fully"). For VE_S, time since vaccination is estimated for the household contacts. The estimates of vaccine effectiveness against infectiousness (VE_I) is given as a pooled estimate and stratified by the vaccination status of the contacts within the household. For VE_I, time since vaccination is estimated for the primary cases. Note that the VE estimates across columns are not directly comparable as they are estimated on stratified samples. 95% confidence intervals clustered on the household level in parentheses. FE = included as fixed effects in the model. Note, the negative VE_I estimates, when the household contacts were fully vaccinated, suggest that there is bias in the comparison of the vaccinated and unvaccinated population that we do not fully control for. We did not find negative VE_I estimates, when we conditioned on the household contacts having a test result (Appendix Table S9), indicating that differences in the probability of being tested across unvaccinated and fully vaccinated contacts is a bias in our model (Appendix S2.4). Other behavioral biases across unvaccinated and fully vaccinated individuals are also likely. Thus it is most likely that there is a waning effect of vaccination, but it is very unlikely that the effect is negative.

Table S9: Vaccine effectiveness by time since vaccination, conditional on household contacts having a test result

	Susceptibility			Infectiousness		
Primary cases vaccinated	**Pool**	**Not**	**Fully**			
Household contacts vaccinated				**Pool**	**Not**	**Fully**
Estimator	$VE_{S(\cdot,V/\cdot,N)}$	$VE_{S(N,V/N,N)}$	$VE_{S(V,V/V,N)}$	$VE_{I(V,\cdot/N,\cdot)}$	$VE_{I(V,N/N,N)}$	$VE_{I(V,V/N,V)}$
Time since vaccination						
0-1 months	73	69	69	63	45	49
	(71;75)	(67;71)	(65;73)	(59;66)	(38;51)	(41;56)
2-3 months	55	50	50	45	30	14
	(52;58)	(46;54)	(43;56)	(41;48)	(24;37)	(3;23)
4-5 months	49	45	48	41	29	15
	(44;53)	(36;53)	(39;55)	(35;46)	(15;41)	(2;27)
5-6 months	39	29	37	37	28	0
	(31;46)	(17;40)	(24;48)	(29;44)	(12;40)	(-18;15)
7-8 months	43	30	50	42	30	9
	(29;53)	(8;48)	(31;63)	(31;52)	(11;45)	(-15;28)
Age FE	YES	YES	YES	YES	YES	YES
Week FE	YES	YES	YES	YES	YES	YES
Female	YES	YES	YES	YES	YES	YES
Household size FE	YES	YES	YES	YES	YES	YES
Female, primary case	YES	YES	YES	YES	YES	YES
Age, primary case, FE	YES	YES	YES	YES	YES	YES
Ct value, primary case, FE	NO	NO	NO	NO	NO	NO
Conditional on test	YES	YES	YES	YES	YES	YES
N Observations	42,170	29,472	12,698	42,170	20,592	21,578
N Households	21,086	13,698	7,388	21,086	12,744	14,201

Notes: This table provides VE estimates from the same model as Table 2 by time since vaccination (bi-monthly), conditional on the household contact having a test result. This table provides estimates of vaccine effectiveness (%) against susceptibility (VE_S) as a pooled estimate ("Pool") as well as stratified by whether the primary case was unvaccinated ("Not") or fully vaccinated ("Fully"). For VE_S, time since vaccination is estimated for the household contacts. The estimates of vaccine effectiveness against infectiousness (VE_I) is given as a pooled estimate and stratified by the vaccination status of the contacts within the household. For VE_I, time since vaccination is estimated for the primary cases. Note that the VE estimates across columns are not directly comparable as they are estimated on stratified samples. 95% confidence intervals clustered on the household level in parentheses. FE = included as fixed effects in the model.

S3 Statistical Appendix

This section provides more details of the statistical methods used to generate the results presented in the main manuscript.

We defined the secondary attack rate (SAR) as the proportion of contacts that were infected within each household 1-14 days after exposure to a primary case. We only included individuals that were either fully vaccinated (V) or not vaccinated (N), thus excluding individuals that were partially vaccinated.

To compare attack rates across different vaccination status of primary cases and contacts, we estimated the relative risk (RR). The vaccine effectiveness (VE) is given by one minus the relative risk (1-RR) of the SAR of vaccinated individuals compared to the SAR of the unvaccinated individuals. We stratified our analyses in order to separate the VE of susceptibility to infection of the exposed contact (VE_S) from the VE of infectiousness in infected primary cases (VE_I), and to estimate the total VE (VE_T). In particular, we use the following 7 equations, with underlying estimates for SAR produced using generalized linear models that control for age, sex, household size, and calendar week, with standard errors clustered on the household level.

Let $SAR_{(\theta_p,\theta_i)}$ denote the SAR, where θ is the vaccination status $\theta \in \{V, N\}$ (*Fully Vaccinated* or *Not vaccinated*) of primary cases (p) and contacts (i). Let "$\cdot$" denote the pooled sample of both vaccination statuses. Thus, the pooled SAR is denoted as $SAR_{(\cdot,\cdot)}$

Vaccine effectiveness of susceptibility (VE_S)

First, we compared the SAR across contacts that were fully vaccinated and contacts that were not vaccinated, unconditional of the vaccination status of the primary case.

The pooled VE of susceptibility to infection in exposed contacts (VE_S) is given by:

$$VE_{S(\cdot,V/\cdot,N)} = 1 - \frac{SAR_{(\cdot,V)}}{SAR_{(\cdot,N)}}, \tag{1}$$

i.e., the SAR of vaccinated contacts relative to the SAR of unvaccinated contacts—unconditional on the vaccination status of the primary case.

Next, we hold the vaccination status of the primary case fixed, and again compare the SAR across contacts that are fully vaccinated and not vaccinated.

VE of susceptibility to infection in exposed contacts among unvaccinated primary cases is given by:

$$VE_{S(N,V/N,N)} = 1 - \frac{SAR_{(N,V)}}{SAR_{(N,N)}} \tag{2}$$

VE of susceptibility to infection in exposed contacts among fully vaccinated primary cases is given by:

$$VE_{S(V,V/V,N)} = 1 - \frac{SAR_{(V,V)}}{SAR_{(V,N)}} \tag{3}$$

Vaccine effectiveness of infectiousness (VE_I)

First, we compared the SAR across primary cases that were fully vaccinated and not vaccinated, unconditional of the vaccination status of the household contacts.

The pooled VE of infectiousness in primary cases (VE_I) is given by:

$$VE_{I(V,\cdot/N,\cdot)} = 1 - \frac{SAR_{(V,\cdot)}}{SAR_{(N,\cdot)}} \tag{4}$$

Next, we hold the vaccination status of the contacts fixed, and compare the SAR across primary cases that are fully vaccinated and not vaccinated.

VE of infectiousness in primary cases among unvaccinated contacts is given by:

$$VE_{I(V,N/N,N)} = 1 - \frac{SAR_{(V,N)}}{SAR_{(N,N)}} \tag{5}$$

VE of infectiousness in primary cases among fully vaccinated contacts is given by:

$$VE_{I(V,V/N,V)} = 1 - \frac{SAR_{(V,V)}}{SAR_{(N,V)}} \tag{6}$$

Total vaccine effectiveness (VE_T)

Finally, we compared the SAR, where both the primary case and contacts were fully vaccinated, with the SAR, where both the primary case and contacts were unvaccinated.

The total VE is given by:

$$VE_{T(V,V/N,N)} = 1 - \frac{SAR_{(V,V)}}{SAR_{(N,N)}}, \tag{7}$$

Estimation

We estimated the relative risks for VE_S, VE_I and VE_T using the following generalized linear model (GLM), with a Poisson distribution response and log link function:

$$\begin{aligned} y_{i,p} &\sim \text{Poisson}(\lambda_{i,p}) \\ log(\lambda_{i,p}) &= Vacc + Age_i + Age_p + Sex_i + Sex_p + HouseholdSize_p + Week_t\,, \end{aligned} \tag{8}$$

where $Vacc$ is a binary fixed effect representing one of the following explanatory variables for each model:

- For VE_S, $Vacc$ refers to vaccination status of the contact i.

- For VE_I, $Vacc$ refers to vaccination status of the primary case p.

- For VE_T, $Vacc$ refers to vaccination status of both the primary case and contacts p, i.

Age denotes categorical fixed effects of age group in 10 year intervals, Sex is a binary fixed effect controlling for sex, $HouseholdSize$ denotes categorical fixed effects of the number of household members, and $Week$ is a categorical fixed effect of the calendar week t. Standard errors were clustered on the household level in order to control for within-household correlation in risk.

The GLM was fit using maximum likelihood in SAS. Note that the Poisson distribution is used with binary outcome in order to facilitate the calculation of relative risks.

Difference in Ct values for secondary cases

First, to investigate the dynamics of the difference in the Ct values across vaccinated and unvaccinated secondary cases, we estimated the difference in the Ct values across each day since exposure to the primary case, using the following equation:

$$Ct_{i,t} = \sum_{\tau} I_{\tau} + \sum_{\tau} I_{\tau} \times Vacc_i + Age_i + \varepsilon_{i,t}\,, \tag{9}$$

where τ is the time between the secondary case (i) and primary case (p) testing positive, which runs from 1 to 14; I_{τ} is a vector of indicators of time since the primary case. $Vacc_i$ is a dummy for the secondary case being fully vaccinated or unvaccinated. Age_i is a vector of fixed effects for 5 year age groups.

Next, as we found no evidence of an interaction term between time since primary case and vaccination status, we proceeded with the simpler equation to estimate the difference in Ct values across vaccination status of the secondary case:

$$Ct_{i,t} = Vacc_i + \sum_{\tau} I_{\tau} + Age_i + Age_p + Vacc_p + Ct_p + \varepsilon_{i,t}\,, \tag{10}$$

where p denotes the primary case characteristics, and i the secondary case characteristics.
